# Aspirin use for cancer prevention: A systematic review of public, patient and healthcare provider attitudes and adherence behaviours

**DOI:** 10.1016/j.ypmed.2021.106872

**Published:** 2022-01

**Authors:** Kelly E. Lloyd, Louise H. Hall, Natalie King, Rachael J. Thorneloe, Rocio Rodriguez-Lopez, Lucy Ziegler, David G. Taylor, Mairead MacKenzie, Samuel G. Smith, on behalf of the AsCaP Group

**Affiliations:** a Leeds Institute of Health Sciences, University of Leeds, Leeds, UK; b Centre for Behavioural Science & Applied Psychology, Sheffield Hallam University, Sheffield, UK; c School of Pharmacy, University College London, London, UK; d Independent Cancer Patients' Voice, UK

**Keywords:** Preventive therapy, Chemoprevention, Decision-making, Aspirin, NSAID

## Abstract

We undertook a systematic review to synthesise the data on attitudes and behaviour towards the use of aspirin for cancer prevention, and healthcare providers' attitudes towards implementing aspirin in practice. Searches were carried out across 12 databases (e.g. MEDLINE, EMBASE). We used the Mixed Methods Appraisal Tool to evaluate study quality, and conducted a narrative synthesis of the data. The review was pre-registered (PROSPERO: CRD42018093453). Thirty-eight studies were identified. Uptake and adherence data were all from trials. Trials recruited healthy participants, those at higher risk of cancer, and those with cancer. Four studies reported moderate to high (40.9–77.7%) uptake to an aspirin trial among people who were eligible. Most trials (18/22) reported high day-to-day adherence (≥80%). Three trials observed no association between gender and adherence. One trial found no association between adherence and colorectal cancer risk. Three studies reported moderate to high (43.6–76.0%) hypothetical willingness to use aspirin. Two studies found that a high proportion of healthcare providers (72.0–76.0%) perceived aspirin to be a suitable cancer prevention option. No qualitative studies were identified. The likelihood that eligible users of aspirin would participate in a trial evaluating the use of aspirin for preventive therapy was moderate to high. Among participants in a trial, day-to-day adherence was high. Further research is needed to identify uptake and adherence rates in routine care, the factors affecting aspirin use, and the barriers to implementing aspirin into clinical care.

## Highlights

• Most eligible people offered participation in an aspirin trial accepted.
• Among participants, most reported high (≥80%) adherence on a day-to-day basis.
• No studies examined uptake and adherence in routine care.
• Further research is needed to identify the barriers to using aspirin in practice.

## 1 Introduction

Cancer is the second leading cause of death globally (Naghavi et al., 2017), with an estimated 9.6 million cancer deaths worldwide in 2018 (Bray et al., 2018). There is increasing interest in preventive therapy as part of cancer control efforts (Steward and Brown, 2013). A meta-analysis of 45 observational studies found aspirin to be associated with a reduced risk of developing colorectal (relative risk: 0.73, 95% CI = 0.69–0.78) and other gastrointestinal cancers (range, relative risks: 0.61–0.78) (Bosetti et al., 2020). Reviews have also examined the relationship between aspirin and cancer by synthesising the results of randomised controlled trials (RCTs) investigating aspirin for vascular disease prevention. These results showed that individuals taking aspirin, versus no aspirin, had a reduced 20-year risk of developing colon cancer (hazard ratio: 0.76, 95% CI = 0.60–0.96) (Rothwell et al., 2010), and a reduced risk of colorectal cancer death at 10–20 years (hazard ratio: 0.51, 95% CI = 0.35–0.74) (Rothwell et al., 2011). Cohort studies have observed weaker significant associations between aspirin use and risk reduction of non-gastrointestinal cancers, such as breast (hazard ratio: 0.96, 95% CI = 0.91–1.00) (Hurwitz et al., 2021), prostate (hazard ratio: 0.95, 95% CI = 0.90–1.00) (Hurwitz et al., 2021), and lung cancer (relative risk: 0.95, 95% CI = 0.91–0.98) (Jiang et al., 2021).

Despite many countries having national cancer screening programmes, few have implemented guidance recommending aspirin for cancer prevention. The US Preventive Services Taskforce recommends aspirin for colorectal cancer prevention among adults aged 50–69 who have ≥10% 10-year cardiovascular disease risk (Bibbins-Domingo, 2016). In the UK, the National Institute of Health and Care Excellence recommends daily aspirin for people with Lynch syndrome (National Institute for Health and Clinical Excellence (NICE), 2020), and in Australia aspirin is recommended for the public aged 50–70 (Cancer Council Australia, 2019). Guideline implementation depends on informed uptake, high adherence, and understanding the barriers to achieving these goals. However, deciding whether to use preventive therapy can be a complex choice for patients, and for their healthcare providers prescribing it. The benefits of aspirin need to be considered in relation to its side-effects, as even low doses can increase the risk of gastrointestinal bleeding, ulcers and, in more rare cases, haemorrhagic stroke (Lanas and Scheiman, 2007; Cuzick et al., 2015).

Studies have investigated the barriers and facilitators to using breast cancer preventive therapy. The evidence suggests the factors associated with increased uptake include having children (Hackett et al., 2018), higher objective risk (Smith et al., 2016), higher cancer-related worry (Bober et al., 2004; Holmberg et al., 2017), and fewer concerns about the side-effects (Bober et al., 2004; Thorneloe et al., 2019; Rondanina et al., 2008). Women with lower educational qualifications, depression and those who are older are also less likely to adhere to the medication (Smith et al., 2016). Prospective studies have also identified a positive association between healthcare provider recommendation and patients' use of breast cancer preventive therapy (Bober et al., 2004; Holmberg et al., 2017). To our knowledge, no review has examined decision-making in the context of aspirin for cancer prevention among potential users of aspirin and healthcare providers.

We undertook a systematic review to synthesise the quantitative and qualitative data on uptake and adherence behaviours related to aspirin for cancer prevention, investigate the factors affecting decisions to use aspirin, and examine healthcare providers' attitudes towards implementing aspirin in clinical care.

## 2 Materials and methods

### 2.1 Search strategy

We first conducted a search of the literature in March 2018, and reran the searches in February 2020. Searches were conducted in the following databases from inception to February 2020: MEDLINE; EMBASE; CINAHL; Cochrane Library (CENTRAL and Cochrane Database of Systematic Reviews); Database of Abstracts of Reviews of Effects (DARE); NHS Economic Evaluation Database; Pan Health Technology Assessment (HTA) Database; HTA Database (Wiley); PubMed; ProQuest Dissertation and Theses A&I; and Web of Science Core Collection. We also searched the International Clinical Trials Registry Platform (ICTRP) and Clinical trials.gov, and the websites of Cancer Research UK and cancer.gov for any ongoing trials. After identifying relevant conference abstracts, trials, and dissertations, we searched for the peer-reviewed articles of these studies. Search terms were developed for the concepts: aspirin, cancer and prevention by an information specialist (RR) and project team members using subject headings and free text terms (see supplementary appendix for search strategies). We did not apply date limits or methodological filters to the searches.

We stored and de-duplicated the records in EndNote X9, and screened them using the management software Covidence. To find additional papers, we searched the reference lists of included studies and relevant reviews. The review was pre-registered (PROSPERO number: CRD42018093453), and PRISMA guidelines for reporting were followed throughout (Moher et al., 2009).

### 2.2 Study selection

We included both quantitative and qualitative peer-reviewed studies, which provided empirical data and recruited individuals aged 18 or over. Studies were included if they reported rates of uptake and/or adherence to aspirin (at any dose) for primary or secondary prevention (i.e. preventing recurrence) of cancer. Additionally, we included articles which reported patient, public or healthcare provider attitudes towards using aspirin for cancer prevention. We deviated from the pre-registration by including quantitative studies exploring individuals' perceptions about taking aspirin for cancer prevention, instead of only qualitative data. Articles on the same trial were included if they provided additional data, such as adherence at longer follow up. We excluded articles reporting adherence on a smaller sub-sample from an included trial.

As we were only interested in attitudes and behaviour data in the context of aspirin for cancer prevention, we excluded studies where aspirin was not used/prescribed for the primary purpose of cancer prevention. For example, we excluded studies using aspirin for the primary purpose of cardiovascular disease prevention/management, and case control and cohort studies if aspirin was not being used for the primary purpose of cancer prevention. Non-peer reviewed studies and reviews were also excluded. We excluded by hand non-English language studies as we did not have the resources to translate.

Screening of the titles and abstracts was completed by two authors (RJT, KEL), and two authors (RR, LHH) duplicated screening for 20% of articles. Discrepancies were resolved with a third reviewer (SGS). Two authors (RJT, KEL) screened the full text articles, and second reviewers (LHH, KEL) duplicated screening for 20% of articles. The review was managed in Covidence.

### 2.3 Data extraction

Two authors (RJT, KEL) extracted the study data using Excel, and 45% (17/38) of a random sample of articles were verified by second reviewers (RR, KEL) to ensure consistency (Shea et al., 2017). We extracted data on study characteristics; sample characteristics; aspirin dose; timing; uptake level; adherence method; adherence definitions; follow-up time; day-to-day adherence; persistence adherence; and factors associated with uptake, day-to-day adherence and/or persistence. Additionally, we extracted data reporting attitudes towards aspirin for cancer prevention.

Uptake rates were defined as the proportion of individuals who were offered aspirin and took the first dose (Vrijens et al., 2012). To calculate uptake to a clinical trial, we calculated the proportion of eligible participants who enrolled on the trial. The denominator was the number of eligible participants offered the trial, with ineligible participants excluded from the calculation. We classified participants who declined trial participation for unknown reasons as declining to take part. We defined day-to-day adherence as the extent to which people took the medication as prescribed (Vrijens et al., 2012). Data could be continuous (0–100% of medications) or categorical (proportion classified as adherent). We defined persistence as the length of time between uptake and last dose (Vrijens et al., 2012). Studies reporting the proportion of participants who completed the trial, without explicit reference to the medication, were excluded. We included both self-report and objective adherence measures.

### 2.4 Quality assessment

We used the Mixed Method Appraisal Tool (MMAT) to assess methodological quality (Pluye and Hong, 2014). MMAT is reliable (Pace et al., 2012), and has been used in a review examining decision-making in breast cancer preventive therapy (Smith et al., 2016). For each study design (qualitative, quantitative RCTs, non-randomised quantitative studies, quantitative descriptive studies, mixed methods studies), there was a quality checklist consisting of 5 items. All items were categorised as ‘Yes’, ‘No’, or ‘Can't tell’.

RCTs received a quality assessment score ranging from 0 to 4, as the criterion ‘Did the participants adhere to the assigned intervention?’ (2.5) was removed due to adherence being a review outcome. All other study types received a score 0–5. The MMAT guidance recommended study teams agreed on an acceptable dropout rate for the criterion ‘Are there complete outcome data?’ (2.3, 3.3). We decided a priori that an article would qualify as ‘Yes’ if they reported a dropout rate of ≤30% participants (Pluye and Hong, 2014; Furlan et al., 2009). One author (KEL) assessed the quality of all articles, with over 35% (14/38) of a random sample of articles verified by a second author (LHH) to ensure consistency. Any discrepancies were resolved with a third author (SGS).

### 2.5 Synthesis of the evidence

To determine if a meta-analysis was appropriate we considered whether the included studies were sufficiently similar on the domains of participants (setting), intervention, comparison and outcomes (Ryan and Cochrane Consumers and Communication Review Group, 2016). There was substantial heterogeneity, for example there was high variations in the doses of aspirin prescribed (intervention), assessments of adherence (outcomes), and the participant population (setting). Within subgroups, few studies used the same setting, intervention, and outcome. Therefore, we concluded that a meta-analysis was inappropriate for our review due to the high heterogeneity. Instead we conducted a narrative synthesis, with findings tabulated (Petticrew and Roberts, 2006). We organised the studies into categories and synthesised the findings (Petticrew and Roberts, 2006). Where possible, comparisons were made between studies on the setting (trial vs. routine care), sample population, aspirin dose/frequency, and healthcare provider population. Across the different categories, we also examined if there was a relationship between year of study, and age of the sample, on the review outcomes.

## 3 Results

**Fig. 1:**
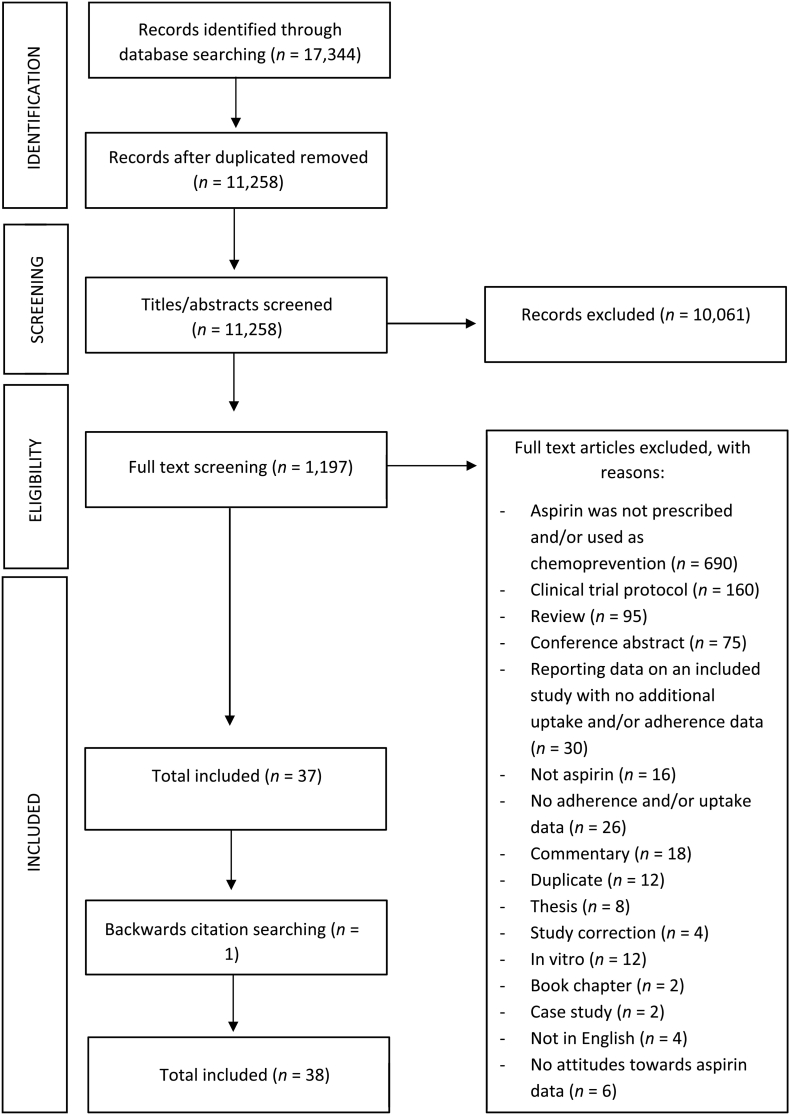
Flow diagram of search strategy.

We identified 17,344 papers, of which 11,258 papers remained after duplicates were removed (Fig. 1). After screening titles and abstracts, we excluded 10,061 articles. We screened 1197 full text articles, 37 studies met the eligibility criteria, and one study was identified by backwards citation searching. A total of 38 studies were included.

### 3.1 Uptake of aspirin

Four studies reported data on uptake of participants to an aspirin clinical trial (Logan et al., 2008; Rexrode et al., 2000; Hull et al., 2018; Jankowski et al., 2018), and all investigated aspirin for primary cancer prevention (Table 1). No studies were identified reporting uptake rates in routine care. All studies were RCTs (Logan et al., 2008; Rexrode et al., 2000; Hull et al., 2018; Jankowski et al., 2018), and of mixed quality with scores ranging from one (Rexrode et al., 2000) to four (Hull et al., 2018) on the MMAT. Three studies (75%) recruited participants at higher risk of developing cancer (Logan et al., 2008; Hull et al., 2018; Jankowski et al., 2018), and one (25%) recruited a healthy population sample (Rexrode et al., 2000). The dose and frequency of prescribed aspirin varied, from 100 mg every alternative day (Rexrode et al., 2000) to 325 mg administered daily (Jankowski et al., 2018). Rates of uptake among eligible people to an aspirin trial were moderate to high (40.9–77.7%) (Logan et al., 2008; Rexrode et al., 2000; Hull et al., 2018; Jankowski et al., 2018).

**Table 1:** Characteristics of articles reporting uptake rates to a clinical trial involving the use of aspirin for cancer prevention (*n* = 4).

Study	Country	Design and quality	Population	Dose/timing	*n*^*⁎*^	Age, years	Eligible participant trial uptake^⁎⁎^
Hull et al., 2018	UK	RCT *MMAT score:* 4	Higher risk patients with colorectal adenomas	300 mg/daily and/or eicosapentaenoic acid	709	Mean: 65	40.9%
Jankowski et al., 2018	UK and Canada	RCT *MMAT score:* 2	Patients with Barrett's oesophagus	300 mg/daily (UK) or 325 mg/daily (Canada) plus esomeprazole	2557	Mean: 58	77.7%
Logan et al., 2008	UK	RCT *MMAT score:* 3	Higher risk patients with colorectal adenomas	300 mg/daily or 300 mg plus folate/daily	939	Mean (range): 57.8 (27.6–74.6)	65.5%
Rexrode et al., 2000	US	RCT *MMAT score:* 1	Women healthcare providers aged ≥45	100 mg/alternate day plus vitamin E	39,876	45–54 (60.2%); 55–64 (29.5%); >65 (10.3%)	61.2%

Key: RCT = Randomised Control Trial; MMAT = Mixed Methods Appraisal Tool; *n*^*⁎*^ *=* number of participants enrolled at the beginning of the study; Eligible participant trial uptake** = proportion of eligible individuals who enrolled on the trial, excluding participants who were ineligible.

Rates of uptake to an aspirin trial did not appear to increase or decrease over time. For example, the oldest study conducted in 2000 reported an uptake rate of 61.2% (Rexrode et al., 2000), while two studies conducted in 2018 reported uptake rates of 40.9% (Hull et al., 2018) and 77.7% (Jankowski et al., 2018). A trial with a mean sample age of 65 years observed lower rates of uptake among eligible people (40.9%) (Hull et al., 2018), compared with studies with a mean sample age of 58 (65.5–77.7%) (Logan et al., 2008; Jankowski et al., 2018). No studies examined the demographic, psychological or clinical factors associated with uptake. No studies compared different aspirin doses and uptake. See supplementary Table 1 for the proportion of participants who enrolled onto the trial, with the dominator the number of participants offered the trial (i.e. inclusive of ineligible participants).

### 3.2 Adherence to aspirin

A total of 29 studies reported aspirin adherence data (Logan et al., 2008; Hull et al., 2018; Jankowski et al., 2018; Barnes et al., 1999; Burney et al., 1996; Cook et al., 2013; Duggan et al., 2014; Frommel et al., 1997; Krishnan et al., 2001; Lipton et al., 1982; Roop et al., 2013; Roy et al., 2017; Ruffin et al., 1997; Sample et al., 2002a; Sandler et al., 2003; Liesenfeld et al., 2016; Benamouzig et al., 2001; Benamouzig et al., 2003; Benamouzig et al., 2012; Ishikawa et al., 2013; Baron et al., 2003; Burn et al., 2008; Burn et al., 2013; Falk et al., 2012; Pommergaard et al., 2016; Sample et al., 2002b; Garland et al., 2019; Joharatnam-Hogan et al., 2019; Sinicrope et al., 2019), and of these 83% (24/29) were RCTs (Table 2) (Logan et al., 2008; Hull et al., 2018; Jankowski et al., 2018; Cook et al., 2013; Duggan et al., 2014; Lipton et al., 1982; Roop et al., 2013; Roy et al., 2017; Sample et al., 2002a; Sandler et al., 2003; Liesenfeld et al., 2016; Benamouzig et al., 2001; Benamouzig et al., 2003; Benamouzig et al., 2012; Ishikawa et al., 2013; Baron et al., 2003; Burn et al., 2008; Burn et al., 2013; Falk et al., 2012; Pommergaard et al., 2016; Sample et al., 2002b; Garland et al., 2019; Joharatnam-Hogan et al., 2019; Sinicrope et al., 2019). Study quality was mixed according to the MMAT scoring, with 48% (14/29) of studies assessed as medium (3/4 or 3/5) or high (4/5 or 4/4) quality (Logan et al., 2008; Hull et al., 2018; Barnes et al., 1999; Cook et al., 2013; Frommel et al., 1997; Krishnan et al., 2001; Roy et al., 2017; Ruffin et al., 1997; Sandler et al., 2003; Ishikawa et al., 2013; Baron et al., 2003; Burn et al., 2013; Pommergaard et al., 2016; Joharatnam-Hogan et al., 2019). The sample characteristics varied, with nearly half of studies (16/29, 55%) recruiting a population at increased risk of developing cancer (Logan et al., 2008; Hull et al., 2018; Jankowski et al., 2018; Barnes et al., 1999; Sample et al., 2002a; Benamouzig et al., 2001; Benamouzig et al., 2003; Benamouzig et al., 2012; Ishikawa et al., 2013; Baron et al., 2003; Burn et al., 2008; Burn et al., 2013; Falk et al., 2012; Pommergaard et al., 2016; Sample et al., 2002b; Garland et al., 2019), such as patients with colorectal adenomas. Five studies (17%) recruited participants with or who previously had cancer (Frommel et al., 1997; Lipton et al., 1982; Roop et al., 2013; Sandler et al., 2003; Joharatnam-Hogan et al., 2019), and five (17%) studies recruited healthy populations (Burney et al., 1996; Cook et al., 2013; Duggan et al., 2014; Ruffin et al., 1997; Liesenfeld et al., 2016). Three studies recruited mixed populations (e.g. higher risk, general public) (Krishnan et al., 2001; Roy et al., 2017; Sinicrope et al., 2019). Most studies investigated aspirin for gastrointestinal cancer prevention, however five studies (17%) examined the relationship between aspirin and the prevention of non-gastrointestinal cancers. These were lung (Cook et al., 2013; Garland et al., 2019), breast (Cook et al., 2013; Duggan et al., 2014; Roop et al., 2013; Joharatnam-Hogan et al., 2019), and prostate cancer (Joharatnam-Hogan et al., 2019).

**Table 2:** Characteristics of articles reporting adherence to aspirin for cancer prevention (*n* = 29).

Study and location	Design and quality	Population	Dose/timing	*n*^*⁎*^	Age, years	Adherence measure	Day-to-day adherence definition	Persistence adherence definition	Follow-up time	Day-to-day adherence	Persistence adherence	Associations with adherence/ persistence
Barnes et al., 1999 US	Non- randomised *MMAT score:* 4	Adenomatous polyps	81 mg/daily	10	Mean (range) 53.6 (47–64)	Pill count	% who took medication	–	3 months	100.0%	–	None reported
Baron et al., 2003 US and Canada	RCT *MMAT score:* 3	Colorectal adenomas	81 mg/daily or 325 mg/daily	1121	Mean (SD): 57.3 (9.9) - 57.7 (9.1)	Self-report	% who took 6–7 tablets/week	% who took ≥50% tablets in final year of trial	Approx. 3 years	81 mg aspirin: 89.8% 325 mg aspirin: 88.0% Placebo: 87.1%	Year 1: 97.8% Year 3: 93.6%	None reported
Benamouzig et al., 2001 France	RCT *MMAT score:* 1	Colorectal adenomatous polyps	300 mg/daily or 160 mg/daily	274	Mean (SD) 57.7 (9.4)	Pill count	% of pills taken	–	16 months	84.1%	–	No association with risk (ND)^+^ No association with gender (ND)^+^
Benamouzig et al., 2003 France	RCT *MMAT score:* 1			272			Mean % of pills taken	–	Approx. 1 year	Aspirin: 87.0% Placebo: 88.0%	–	No association with risk (ND)^+^ No association with gender (ND)^+^
Benamouzig et al., 2012 France	RCT *MMAT score:* 2						Mean % of pills taken	–	Approx. 4 years	88.0%	–	Adherence similar between aspirin 160 mg/day vs. aspirin 300 mg/day vs. placebo (ND)^+^
Burn et al., 2008 International	RCT *MMAT score:* 2	LS	300 mg/twice daily plus resistant starch	937	Mean (range): 45 (25–79)	Pill count	% who took the tablets ≥80.0% of the time	–	Approx. 2 years	81.0%	–	None reported
Burn et al., 2013 International	RCT *MMAT score:* 3						% who took 1400 (300 mg) pills ≥2 years	Mean duration of treatment		Aspirin: 30.0% Placebo: 29.1%	Mean: 25.2 months	None reported
Burney et al., 1996 US	Non-randomised *MMAT score:* 2	Healthy adults	Up to 640 mg/daily	64	Not reported	Self-report and MEMS	% who took ≥80.0% of the pills	–	14 days	Self-report: 73.0% MEMS: 44.0% Self-report and MEMS: 35.0%	–	Self-report vs. MEMS (*p* = 0.002) No association with gender (*p* = 0.95, *p* = 0.78)
Cook et al., 2013 US	RCT *MMAT score:* 3	Healthy female healthcare providers	100 mg/alternate day Plus vitamin E	39,876	Mean: 55	Self-report	Active trial: % took ≥2/3 of aspirin Post-trial: % took aspirin ≥3 days per month	Median duration of treatment	Active trial: 8 years Post-trial: 15 years	Active trial: Aspirin (64.0%) Placebo (65.0%) Post-trial: Aspirin (46.0%) placebo (43.0%)	Median: 9 years	None reported
Duggan et al., 2014 US	RCT *MMAT score:* 2	Post-menopausal women	325 mg/daily	144	Mean (SD): 59.4 (5.4)	Pill count	% of pills taken	–	6 months	Aspirin (87.0%) placebo (87.0%)	–	None reported
Falk et al., 2012 US, Canada, Puerto Rico	RCT *MMAT score:* 2	Barrett's Oesophagus	81 mg/daily or 325 mg/daily Plus esomeprazole	122	Mean (SD): 59.7 (11.2)	Not reported	Median number of tablets taken Percentage of adherence (median)	–	28 days	27–28 tablets for aspirin and placebo (median) 100.0% (median)	–	None reported
Frommel et al., 1997 US	Non-randomised *MMAT score:* 3	CRC	325 mg/daily then 325 mg/twice daily	17	Mean (SD): 65.6 (13.6)	Bleeding time	–	Increase in bleeding time at 120 days	120 days	–	94.1%	None reported
Garland et al., 2019 US	RCT *MMAT score:* 2	High risk of lung cancer	Intermittent: 81 mg/daily one week/placebo one week Continuous: 81 mg/daily	54	Mean (SD): 52 (8)	Pill count	Mean % of pills taken	% who completed the intervention	12 weeks	98.0%	83.3%	None reported
Hull et al., 2018 UK	RCT *MMAT score:* 4	Colorectal adenomas	300 mg/daily and/or eicosapentaenoic acid	709	Mean: 65	Self-report	Mean % of pills taken	–	1 year	Aspirin: 97.0% Placebo: 97.0%	–	None reported
Ishikawa et al., 2013 Japan	RCT *MMAT score:* 3	FAP	100 mg/daily	34	Mean (SD): 36.7 (13.9) – 39.7 (12.8)	Self-report	Not reported	–	10 months	Aspirin: 83.3% Placebo: 88.4%	–	None reported
Jankowski et al., 2018 UK and Canada	RCT *MMAT score:* 2	Barrett's oesophagus	300 mg/daily (UK) or 325 mg/daily (Canada) Plus esomeprazole	2557	Mean: 58–59	Not reported	–	% still taking aspirin at 10 years Median duration of treatment	Approx. 10 years	–	>25% still taking aspirin at 10 years Median: 8.9 years	None reported
Joharatnam-Hogan et al., 2019 UK	RCT *MMAT score:* 3	Gastro-oesophageal, CRC, breast, prostate cancer	100 mg/daily or 300 mg/daily	2719	Median: 52–71	Self-report	% who took 6–7 tablets/week	–	8 weeks	95.0%	–	None reported
Krishnan et al., 2001 US	Non-randomised *MMAT score:* 4	High vs. normal risk for CRC	81 mg/daily	92	Mean (SD): 36.5 (14.8) – 55.2 (13.9)	Self-report and pill counts	% who took ≥80.0% of the pills	–	28 days	100.0%	–	None reported
Liesenfeld et al., 2016 US	RCT *MMAT score:* 2	Healthy men and women	325 mg/daily	40	Mean (SD): 31 (6.2)	Salicylic acid metabolites	–	% with salicylic acid metabolites detected at study end	60 days	–	92.5%	None reported
Lipton et al., 1982 US	RCT *MMAT score:* 1	Dukes B_2_ and CRC/rectal cancer	600 mg/twice daily	66	Not described	Blood salicylate levels	–	% who had a salicylate level of ≥4 mg/dl at study end	Not described	–	83.3%	None reported
Logan et al., 2008 UK	RCT *MMAT score:* 3	Colorectal adenomas	300 mg/daily or 300 mg plus folate/daily	939	Mean (range): 57.8 (27.6–74.6)	Self-report and pill count	% who took ≥95.0% of the pills	% who completed trial medication	Approx. 3 years	Aspirin: 75.4% Placebo: 76.4%	66.8%	None reported
Pommergaard et al., 2016 International	RCT *MMAT score:* 3	Colorectal adenomas	37.5 mg aspirin with calcium carbonate/twice daily Plus calcitriol	1107	Median (SD): 59 (8.1) – 60 (8.3)	Pill count	Median % of pills taken	% who completed 3 years of treatment	3 years	Aspirin: 99.0% Placebo: 99.0%	38.6%	None reported
Roop et al., 2013 US	RCT *MMAT score:* 2	Metastatic breast cancer	325 mg/daily plus clopidogrel	48	Mean: 50.7–58.4	Platelet-function tests	–	Inhibition of platelet-function	4 weeks	–	*p* < 0.001	None reported
Roy et al., 2017 US	RCT *MMAT score:* 3	Colonoscopy For adenoma or CRC resected	325 mg/daily	79	Mean (SD): 54 (11) – 57 (9)	Clinical assessment and pill counts	% who took ≥80.0% of the pills	–	3-months	Aspirin: 100.0% Placebo: 100.0%	–	None reported
Ruffin et al., 1997 US	Non-randomised *MMAT score:* 3	Healthy participants	40.5 mg, 81 mg, 162 mg, 324 mg, or 648 mg/daily	66	Mean (range) 27.8 (19–56)	Self-report and MEMS	% who took an extra dose on day 15	% who completed the protocol	14 days	40.5 mg = 20.0% 81 mg = 10.0% 162 mg = 20.0% 324 mg = 10.0%	98.5%	None reported
Sample et al., 2002a US	RCT *MMAT score:* 1	Colorectal adenomas	81 mg/daily or 325 mg/daily or 650 mg/daily	60	Mean: 58.2	Self-report, pill count; plasma salicylate levels	% of pills taken	% whose plasma salicylate levels significantly exceeded baseline	4 weeks	99.0%	93.0% (81 mg); 100.0% (325 mg); 79.0% (650 mg)	None reported
Sample et al., 2002b US	RCT *MMAT score:* 1			43	40–50 (10.5%); 51–60 (36.8%); 61–70 (52.6%)	Self-report	–	% taking aspirin regularly at mean 17.3 months	–	–	41.9%	None reported
Sandler et al., 2003 US	RCT *MMAT score:* 3	CRC	325 mg/daily	635	≤39 (1%); 40–49 (14%); 50–59 (24%); 60–69 (33%); ≥70 (28%)	Self-report	% taking 7 pills per week	Median duration of treatment	Not reported	–	Median: 30.9 months	None reported
Sinicrope et al., 2019 US	RCT *MMAT score:* 2	Advanced adenomas or cancer	325 mg/daily plus Difluoromethylornithine	104	Mean (SD): 62.6 (9.09)	Pill count	% who took ≥80.0% of the pills	–	1 year	98.1%	–	None reported

Key: RCT = Randomised Control Trial; *n*^*⁎*^ *=* number of participants enrolled at the beginning of the study; ND = no data presented; ^+^significance testing not reported; MEMS = Medication Event Monitoring System; FAP = Familial Adenomatous Polyposis; LS = Lynch Syndrome; CRC = Colorectal Cancer.

There was high heterogeneity across the studies, with multiple definitions of day-to-day adherence, ranging from the proportion who took ≥80% of aspirin (Burney et al., 1996; Krishnan et al., 2001; Roy et al., 2017; Ruffin et al., 1997; Burn et al., 2008; Falk et al., 2012), and percentage of pills taken (Hull et al., 2018; Duggan et al., 2014; Sample et al., 2002a; Benamouzig et al., 2001; Benamouzig et al., 2003; Benamouzig et al., 2012; Pommergaard et al., 2016; Garland et al., 2019). Doses of aspirin were administered from 40.5 mg daily (Ruffin et al., 1997) to 600 mg twice daily (Lipton et al., 1982). Adherence measures varied, with 15 out of 29 studies (52%) using objective measures (e.g. pill count, Medication Event Monitoring System (MEMS)) (Barnes et al., 1999; Duggan et al., 2014; Frommel et al., 1997; Lipton et al., 1982; Roop et al., 2013; Roy et al., 2017; Liesenfeld et al., 2016; Benamouzig et al., 2001; Benamouzig et al., 2003; Benamouzig et al., 2012; Burn et al., 2008; Burn et al., 2013; Pommergaard et al., 2016; Garland et al., 2019; Sinicrope et al., 2019). Seven studies (24%) used self-report measures (Hull et al., 2018; Cook et al., 2013; Sandler et al., 2003; Ishikawa et al., 2013; Baron et al., 2003; Sample et al., 2002b; Joharatnam-Hogan et al., 2019), and five studies (17%) used a combination of self-report and objective measures (Logan et al., 2008; Burney et al., 1996; Krishnan et al., 2001; Ruffin et al., 1997; Sample et al., 2002a). Two studies did not report their adherence measurement (Jankowski et al., 2018; Falk et al., 2012).

Day-to-day adherence estimates varied (30.0–100.0%), however 82% (18/22 studies) reported high adherence rates of aspirin (≥80.0% adherence levels) (Hull et al., 2018; Barnes et al., 1999; Duggan et al., 2014; Krishnan et al., 2001; Roy et al., 2017; Ruffin et al., 1997; Sample et al., 2002a; Benamouzig et al., 2001; Benamouzig et al., 2003; Benamouzig et al., 2012; Ishikawa et al., 2013; Baron et al., 2003; Burn et al., 2008; Falk et al., 2012; Pommergaard et al., 2016; Garland et al., 2019; Joharatnam-Hogan et al., 2019; Sinicrope et al., 2019). High levels of day-to-day adherence (≥80.0%) were observed across studies using self-report measures (Hull et al., 2018; Ishikawa et al., 2013; Baron et al., 2003; Joharatnam-Hogan et al., 2019) and those using objective adherence measures (Barnes et al., 1999; Duggan et al., 2014; Benamouzig et al., 2001; Benamouzig et al., 2003; Benamouzig et al., 2012; Burn et al., 2008; Pommergaard et al., 2016; Garland et al., 2019; Sinicrope et al., 2019). Four studies reported on day-to-day adherence three to four years after participants started aspirin (Logan et al., 2008; Benamouzig et al., 2012; Baron et al., 2003; Pommergaard et al., 2016). Of these studies, three observed high adherence levels (≥80%) (Benamouzig et al., 2012; Baron et al., 2003; Pommergaard et al., 2016). One RCT reported data on healthy participants for eight years in the active trial, and for 15 years post-trial (Cook et al., 2013). At eight years, 64.0% of participants were classed as adherent (Cook et al., 2013). By 15 years, 46.0% were adherent (Cook et al., 2013). No pattern was observed between participants' age and day-to-day adherence.

There was no clear evidence of a relationship between dose and day-to-day adherence. In an RCT of high-risk participants, lower adherence was reported among those taking 650 mg of aspirin (79.0% adherent), compared with those taking aspirin at 325 mg (100.0% adherent) and 81 mg (93.0% adherent) (Sample et al., 2002a). Three other studies reported adherence rates across different doses of aspirin and identified few differences (Benamouzig et al., 2012; Baron et al., 2003; Falk et al., 2012). We also observed no pattern between when the study was conducted (older vs. newer studies) and day-to-day adherence.

Persistence was reported by 52% (15/29) of studies (Logan et al., 2008; Jankowski et al., 2018; Cook et al., 2013; Frommel et al., 1997; Lipton et al., 1982; Roop et al., 2013; Ruffin et al., 1997; Sample et al., 2002a; Sandler et al., 2003; Liesenfeld et al., 2016; Baron et al., 2003; Burn et al., 2013; Pommergaard et al., 2016; Sample et al., 2002b; Garland et al., 2019). Measurements of persistence varied from average number of months/years participants were taking the medication (Jankowski et al., 2018; Cook et al., 2013; Sandler et al., 2003; Burn et al., 2013), to increase in bleeding time (Frommel et al., 1997). Short-term persistence (i.e. weeks, months) was high (83.3–100.0%) (Frommel et al., 1997; Ruffin et al., 1997; Sample et al., 2002a; Liesenfeld et al., 2016; Garland et al., 2019). The proportion of participants reporting long-term persistence (i.e. years) varied. Three RCTs, all recruiting participants with colorectal adenomas, examined persistence at three years (Logan et al., 2008; Baron et al., 2003; Pommergaard et al., 2016). One RCT observed high levels of persistence, with 93.6% of participants still taking at least 50% of the medication at year three (Baron et al., 2003). In contrast, two trials reported low to moderate levels of persistence, with 38.6% and 66.8% of participants completing the three-year medication (Logan et al., 2008; Pommergaard et al., 2016). No pattern was observed between the year the study was conducted and persistence with aspirin. Additionally, no pattern was observed between participants' age and persistence with aspirin. For example, both a trial with a mean sample age of 31 (Liesenfeld et al., 2016), and a trial with a mean age of 66 reported high levels of persistence (≥90%) (Frommel et al., 1997).

Four studies examined factors associated with day-to-day adherence. A non-randomised trial of healthy participants found self-report measures to be significantly associated with higher adherence (73.0% adherent), than the objective measure of MEMS (44.0% adherent) (Burney et al., 1996). Two RCTs and one non-randomised trial observed no association between adherence and gender (Burney et al., 1996; Benamouzig et al., 2001; Benamouzig et al., 2003). In an RCT of participants with history of colorectal adenomas, no association was found between adherence and being at higher risk of recurrence, when compared with those at lower risk (Benamouzig et al., 2001). No other factors associated with day-to-day adherence or persistence were reported.

### 3.3 Attitudes towards the use of aspirin for cancer preventive therapy

#### 3.3.1 High risk and general public

Five quantitative descriptive studies examined individuals' attitudes towards using aspirin for the primary prevention of cancer (Yachimski et al., 2015; Jensen et al., 2016; Hur et al., 2009; Hur et al., 2008; Nguyen et al., 2019) (Table 3). All studies were of low (2/5) or medium (3/5) quality, and all were cross-sectional surveys. Three studies (60%) recruited healthy population samples (Jensen et al., 2016; Hur et al., 2009; Nguyen et al., 2019), and two studies (40%) recruited patients with Barrett's oesophagus (Yachimski et al., 2015; Hur et al., 2008). Four studies reported moderate to high willingness from participants to use aspirin for cancer prevention (43.6–76.0%) (Yachimski et al., 2015; Hur et al., 2009; Hur et al., 2008; Nguyen et al., 2019).

**Table 3:** Characteristics of articles reporting public, patient and healthcare provider attitudes towards using or recommending aspirin for cancer prevention (*n* = 8).

Study and location	Design and quality	Population	Setting	Outcomes	*n*^*⁎*^	Age, years	Attitudes towards aspirin for cancer prevention	Associations with higher attitudes (e.g. willingness, intentions)
Chen et al., 2017 Australia	Cross-sectional survey *MMAT score:* 3	Clinicians (genetics providers; gastroenterologists; colorectal surgeons)	HCP survey	- discuss aspirin for cancer prevention with patients - recommends/ prescribes aspirin to patients	181	<50 (60.0%) ≥50 (40.0%)	76.0% thought aspirin was ‘somewhat’ or ‘very’ effective	Univariable analysis: - professional group Multivariable analysis: - no association
Das et al., 2008 UK	Cross-sectional survey *MMAT score:* 3	Gastroenterologists	HCP survey	Variation in practice of BO management	226	ND	72.0% thought using aspirin or COX-2 was a good option	None reported
Hur et al., 2008 US	Cross-sectional survey *MMAT score:* 2	BO patients	Patient survey	Patient preferences for celecoxib and aspirin for cancer prevention	100	Mean (SD): 64.5 (11.3)	76.0% willing to use aspirin	Univariable analysis: - younger age - more educational qualifications Multivariable analysis: - no association
Hur et al., 2009 US	Cross-sectional survey *MMAT score:* 2	Healthy population	Public survey	Patient preferences for celecoxib and aspirin for cancer prevention	202	Median age group: 45–54	43.6% willing to use aspirin	Males (58.1%) more willing to take aspirin than females (31.2%)
Jensen et al., 2016 US	Cross-sectional survey *MMAT score:* 3	Healthy population (aged 40–65)	Public survey	Intentions to use aspirin for cancer prevention on 5-point scale from strongly disagree (1) to strongly agree (5)	1000	Mean (SD): 56.65 (6.87)	Intentions to use aspirin for cancer prevention (M = 3.34, SD = 1.22)	**Demographic variables:** - older - male - black ethnicity **Clinical factors:** - did not already take aspirin - history of polyps - smoked >100 cigarettes **Psychosocial variables:** - increased perceived susceptibility, barriers, response and self-efficacy - reporting less Cancer information overload
Nguyen et al., 2019 Australia	Cross-sectional survey *MMAT score:* 3	Healthy population (aged 50–70)	Public survey	Whether they would take aspirin for bowel cancer prevention	304	50–54 (24.7%) 55–59 (29.6%) 60–64 (21.1%) 65–70 (24.7%)	>70.0% would take aspirin	Current aspirin use (*p* < 0.001) No differences across demographic factors (gender, age, education, martial status), or other clinical factors (family history of CRC)
Smith et al., 2017 UK	Cross-sectional survey *MMAT score:* 3	GPs practising in the UK	HCP survey	Willingness to prescribe LS patients aspirin at 600 mg	1007	<50 (72.3%) ≥50 (27.7%)	62.3% willing to prescribe aspirin at 600 mg	- ≥50 years old - >10 years' experience - without special interest in family history - greater awareness of preventive effects aspirin - having seen LS patient in practice
Yachimski et al., 2015 US	Cross-sectional survey *MMAT score:* 2	BO patients	Patient survey	Willingness to undergo treatment A (ablation) and/or treatment B (aspirin)	81	Mean: 60.2	53.0% willing to use aspirin (with endoscopic surveillance every 3–5 years)	No differences across demographic factors (gender, age, education, ethnicity) and clinical variables (already taking aspirin, using PPI, personal history of cancer, heart condition, and peptic ulcer)

Key: *n*^*⁎*^ = number of participants who took part in the study; HCP = Healthcare provider; GP = General Practitioner; LS *=* Lynch Syndrome; BO = Barrett's Oesophagus; PPI = Proton Pump Inhibitor; CRC = Colorectal Cancer; ND = No Data.

Mixed results were observed for an association between participants' demographic characteristics and whether they would use aspirin for cancer prevention. A US survey examined the relationship between healthy participants' characteristics and intentions to use aspirin (Jensen et al., 2016). Higher intentions were significantly associated with being male, black ethnicity, older age, history of polyps, and being a smoker (Jensen et al., 2016). Another survey recruiting Barrett's oesophagus patients found higher education and younger age to be significantly associated with higher willingness to use aspirin in the univariable analysis (Hur et al., 2008). However, this association was not significant in the multivariable analysis (Hur et al., 2008). Two studies also found no evidence of a relationship between demographic factors and willingness to use aspirin (Yachimski et al., 2015; Nguyen et al., 2019). Mixed evidence was also observed for the relationship between participants' current aspirin use and whether they would use aspirin for cancer prevention (Jensen et al., 2016; Nguyen et al., 2019).

Participants with increased self-efficacy, response efficacy, barriers and perceived susceptibility to developing colorectal cancer were significantly more likely to report higher intentions to use aspirin (Jensen et al., 2016). Some of the barriers found to be significantly and positively associated with intentions included participants' believing their doctor would want them to take aspirin, and believing most people their age were being told to take aspirin (Jensen et al., 2016). Participants who believed there was low evidence for using aspirin for cancer prevention reported significantly lower intentions (Jensen et al., 2016).

No clear relationship was observed between year of study and attitudes towards aspirin. Two papers examined publics' willingness to use aspirin, with the 2019 Australian study finding higher willingness (>70%) (Nguyen et al., 2019), than a US-based study conducted in 2009 (43.6%) (Hur et al., 2009). However, among two US studies, one conducted in 2008 found higher willingness among patients with Barrett's oesophagus (76.0%) (Hur et al., 2008), compared with a 2015 study examining willingness among the same patient population (53.0%) (Yachimski et al., 2015).

#### 3.3.2 Healthcare providers

Three studies reported healthcare providers' attitudes towards aspirin for cancer prevention (Smith et al., 2017; Chen et al., 2017; Das et al., 2008) (Table 3). All studies were of medium MMAT quality (3/5). Samples consisted of gastroenterologists (Chen et al., 2017; Das et al., 2008), genetics professionals (Chen et al., 2017), colorectal surgeons (Chen et al., 2017) and general practitioners (Smith et al., 2017). Two studies reported data on healthcare providers' attitudes towards the use of aspirin for patients at higher risk of cancer (Lynch syndrome, Barrett's oesophagus) (Chen et al., 2017; Das et al., 2008). In both studies, a high proportion of healthcare provider respondents (72.0–76.0%) perceived aspirin to be a suitable cancer prevention option (Chen et al., 2017; Das et al., 2008).

A UK survey of general practitioners found willingness to prescribe aspirin was higher at lower doses, with 91.3% willing at 100 mg, 81.8% willing at 300 mg, and 62.3% willing at 600 mg (Smith et al., 2017). General practitioners were significantly more willing to prescribe aspirin at 600 mg if they had >10 years' professional experience, were aged ≥50, had greater awareness of the preventive effects of aspirin, and if they had seen a Lynch syndrome patient in clinic (range, odds ratio: 1.44 to 1.58) (Smith et al., 2017). There was evidence to suggest profession may influence willingness, with general practitioners who had a special interest in family history significantly less willing to prescribe aspirin (odds ratio: 0.41) (Smith et al., 2017). An Australian survey also found that a higher proportion of gastroenterologists (41/49, 83.7%) and genetic professionals (49/59, 83.1%) perceived aspirin to be effective for cancer prevention, than colorectal surgeons (47/73, 64.4%) (Chen et al., 2017). Across all three studies, we did not observe a pattern between year of study and healthcare providers' attitudes towards aspirin for preventive therapy (Smith et al., 2017; Chen et al., 2017; Das et al., 2008).

### 3.4 Study quality

We assessed methodological quality using the MMAT (Table 4). Twenty-five studies were quantitative RCTs (Logan et al., 2008; Rexrode et al., 2000; Hull et al., 2018; Jankowski et al., 2018; Cook et al., 2013; Duggan et al., 2014; Lipton et al., 1982; Roop et al., 2013; Roy et al., 2017; Sample et al., 2002a; Sandler et al., 2003; Liesenfeld et al., 2016; Benamouzig et al., 2001; Benamouzig et al., 2003; Benamouzig et al., 2012; Ishikawa et al., 2013; Baron et al., 2003; Burn et al., 2008; Burn et al., 2013; Falk et al., 2012; Pommergaard et al., 2016; Sample et al., 2002b; Garland et al., 2019; Joharatnam-Hogan et al., 2019; Sinicrope et al., 2019), 8 were quantitative descriptive studies (Yachimski et al., 2015; Jensen et al., 2016; Hur et al., 2009; Hur et al., 2008; Nguyen et al., 2019; Smith et al., 2017; Chen et al., 2017; Das et al., 2008), and five were quantitative non-randomised studies (Barnes et al., 1999; Burney et al., 1996; Frommel et al., 1997; Krishnan et al., 2001; Ruffin et al., 1997). No qualitative studies were identified. Of the RCTs, one study (4%) scored 4/4 for quality (Hull et al., 2018), 36% (9/25) scored 3/4 (Logan et al., 2008; Cook et al., 2013; Roy et al., 2017; Sandler et al., 2003; Ishikawa et al., 2013; Baron et al., 2003; Burn et al., 2013; Pommergaard et al., 2016; Joharatnam-Hogan et al., 2019), and 24% (6/25) of studies met one criterion (Rexrode et al., 2000; Lipton et al., 1982; Sample et al., 2002a; Benamouzig et al., 2001; Benamouzig et al., 2003; Sample et al., 2002b). Of the quantitative non-randomised studies, two studies (40%) scored 4/5 on the MMAT (Barnes et al., 1999; Krishnan et al., 2001), two studies (40%) scored 3/5 (Frommel et al., 1997; Ruffin et al., 1997), and one study (20%) scored 2/5 (Burney et al., 1996). Of the quantitative descriptive studies, 38% (3/8) scored 2/5 on the MMAT (Yachimski et al., 2015; Hur et al., 2009; Hur et al., 2008), and 63% (5/8) scored 3/5 (Jensen et al., 2016; Nguyen et al., 2019; Smith et al., 2017; Chen et al., 2017; Das et al., 2008).

**Table 4:** Mixed Methods Appraisal Tool assessment for the 38 included studies.

		Yes		No		Cannot tell	
	*n*	*n*	%	*n*	%	*n*	%
**2. Quantitative randomised controlled trials**	25						
2.1. Is randomization appropriately performed?		6	24	0	0	19	76
2.2. Are the groups comparable at baseline?		20	80	2	8	3	12
2.3. Are there complete outcome data?		19	76	4	16	2	8
2.4. Are outcome assessors blinded to the intervention provided?		10	40	4	16	11	44
**3. Quantitative non-randomised studies**	5						
3.1. Are the participants' representative of the target population?		4	80	1	20	0	0
3.2. Are measurements appropriate regarding both the outcome and intervention (or exposure)?		5	100	0	0	0	0
3.3. Are there complete outcome data?		4	80	1	20	0	0
3.4. Are the confounders accounted for in the design and analysis?		0	0	1	20	4	80
3.5 During the study period, is the intervention administered (or exposure occurred) as intended?		3	60	1	20	1	20
**4. Quantitative descriptive studies**	8						
4.1. Is the sampling strategy relevant to address the research question?		4	50	3	38	1	13
4.2. Is the sample representative of the target population?		1	13	5	63	2	25
4.3. Are the measurements appropriate?		5	63	2	25	1	13
4.4. Is the risk of nonresponse bias low?		4	50	1	13	3	38
4.5. Is the statistical analysis appropriate to answer the research question?		7	88	0	0	1	13

## 4 Discussion

In this systematic review investigating attitudes and behaviour towards aspirin for preventive therapy, we found moderate to high levels of uptake to an aspirin clinical trial among people who were eligible to participate. A large proportion of participants in trials reported high levels of adherence on a day-to-day basis. At short-term follow up, most people were still taking aspirin for cancer prevention. However, there was mixed evidence observed for long-term persistence with aspirin. Given that aspirin is recommended to be taken regularly for several years for a cancer preventive benefit (Bibbins-Domingo, 2016; National Institute for Health and Clinical Excellence (NICE), 2020), persistence among users of aspirin should be investigated further.

In contrast to the more extensive behavioural research conducted in breast cancer preventive therapy (Hackett et al., 2018; Smith et al., 2016; Bober et al., 2004; Holmberg et al., 2017; Thorneloe et al., 2019; Rondanina et al., 2008), minimal research has examined the factors associated with use of aspirin for cancer prevention. In our review, we only identified four studies reporting any factors associated with adherence, and none with uptake. Additionally, no qualitative studies were identified. Several studies investigated willingness or intention to use aspirin, which was found to be moderately high among members of the public and those at higher cancer risk. The demographic, clinical and psychological factors associated with willingness and intentions were also investigated, but evidence was either limited or conflicting.

While observational studies were eligible, we only identified trials reporting uptake and adherence data, which presents generalisability issues. Trial participants may be more motivated to use aspirin than those in routine care, and frequent follow-ups may have increased adherence rates. Previous research has also observed that people at lower socioeconomic status (Gross et al., 2005) and those from an ethnic minority group (Du et al., 2006) are less likely to participate in cancer trials. Furthermore, the decision to participate in a trial would not have been just a consideration of aspirin, but also other agents being simultaneously investigated. The four trials reporting uptake data were also evaluating esomeprazole, vitamin E, folate, and eicosapentaenoic acid alongside aspirin. Members of the public may be less familiar with these agents, which may have negatively affected their decision to participate in the trial.

In our review, we identified studies conducted across multiple decades (1982 to 2019). However, official guidance recommending the use of aspirin for colorectal cancer prevention has only recently been introduced (2016 onwards) (Bibbins-Domingo, 2016; National Institute for Health and Clinical Excellence (NICE), 2020; Cancer Council Australia, 2019). While we did not find an increase over time in trial uptake and adherence, future trials may observe higher rates of uptake and adherence as official guidance becomes more widely known among the public and healthcare providers. Furthermore, in the future we may observe an increasing trend in positive attitudes towards aspirin for preventive therapy.

Despite searching for studies using aspirin for secondary cancer prevention, most articles investigated aspirin for primary prevention. Our review findings should be applied with caution to a secondary prevention context. Patients who have previously had cancer may have different motivations for taking aspirin than those offered aspirin for primary prevention. Healthcare providers may also have less positive views towards aspirin for secondary cancer prevention, as a lower number of secondary prevention trials have been conducted compared with primary prevention (Langley et al., 2011). However, there is a large ongoing trial in the adjuvant setting (Add-Aspirin trial), which will provide further evidence on the effects of regular aspirin use in patients with non-metastatic breast, colorectal, gastro-oesophageal, and prostate cancer (Coyle et al., 2016).

Relevant studies have been published following our search cut-off date that contribute further to our knowledge in this topic area. Similar to our review findings, the ASPIRED trial, investigating aspirin for colorectal cancer prevention, found that most participants reported high levels of day-to-day adherence to aspirin at dose of 81 mg (79% reported 95–100% adherence) and 325 mg (91% reported 95–100% adherence) (Drew et al., 2020). Furthermore, a recent qualitative study was published exploring healthcare professionals' views on the Australian guidance recommending aspirin for colorectal cancer prevention for the public (Milton et al., 2021).

### 4.1 Directions for future research

Overall, we found that the likelihood that eligible users of aspirin would participate in a trial that requires randomization to aspirin for cancer prevention was between 40.9 and 77.7%. Researchers developing a trial in this area should take these findings into consideration when planning and designing their study. While clinical guidelines in the US, Australia and the UK recommend aspirin for colorectal cancer prevention (Bibbins-Domingo, 2016; National Institute for Health and Clinical Excellence (NICE), 2020; Cancer Council Australia, 2019), it is currently unknown if people initiate and adhere to aspirin in routine care. To date, only studies reporting data on intentions and willingness to use aspirin have been published. As intentions do not always translate into behaviour (Sheeran and Webb, 2016), further research should investigate how people form a decision to initiate and adhere to aspirin for preventive therapy, and the support they may need.

Despite searching for studies investigating aspirin for any cancer prevention, the vast majority of identified studies focused on gastrointestinal cancer risk reduction. As the evidence base is stronger for gastrointestinal cancer prevention, we may expect lower rates of uptake, adherence and acceptability for other cancers (e.g. breast, lung, prostate). Research should investigate further rates of uptake and adherence of, and attitudes towards, aspirin for the prevention of non-gastrointestinal cancers.

Previous research has found higher uptake of breast cancer preventive therapy among women with fewer concerns about its side-effects (Thorneloe et al., 2019; Rondanina et al., 2008). While there are several reported side-effects to using aspirin (Lanas and Scheiman, 2007; Cuzick et al., 2015), it is currently unknown the relationship between participants' side-effects, perceived or experienced, in relation to aspirin and their rates of uptake and adherence. We recommend that future research should investigate the relationship between these factors further.

The recent Australian qualitative study reported that healthcare providers viewed primary care physicians as having the most important role in the implementation of guidance recommending aspirin for cancer prevention (Milton et al., 2021). We recommend that future research aiming to examine decision-making in the context of aspirin for cancer prevention should focus on the primary care setting. In our review, we found moderately high levels of willingness among general practitioners to prescribe aspirin to patients with Lynch syndrome. Factors that may be influencing willingness include the aspirin dose, professional background, and awareness of the cancer preventive benefits of aspirin.

The review had limitations. Due to time and resource constraints, the literature was limited to English language articles, and second reviewers only duplicated screening, data extraction, and quality assessment for a proportion of articles (20–45%). Our review excluded studies that did not use or prescribe aspirin for the primary purpose of cancer prevention, such as the ASPREE trial which had fatal and non-fatal cancer as a secondary endpoint (McNeil et al., 2018). However, in clinical practice consideration to use aspirin is likely to factor in both its use as a form of cancer preventive therapy and other outcomes, such as cardiovascular disease prevention. Uptake rates to a clinical trial were also strongly affected by the approach used to calculate uptake. For example, as reported in supplementary Table 1, when we calculated rates of uptake to a trial with the denominator all people who were approached about the trial, including those who were ineligible to participate, uptake rates were much lower. More standardised and transparent reporting of uptake data is warranted to compare across cohorts.

## 5 Conclusions

Overall, we found that most people who were eligible and offered participation in an aspirin trial accepted. The majority of participants also reported a good level of adherence on a day-to-day basis. We found high levels of short-term aspirin persistence, but evidence was mixed for long-term persistence. No studies examined uptake and adherence in routine care, and minimal research investigated the factors associated with using aspirin. Overall, we found that there is substantial scope for research into the barriers and facilitators to implementing aspirin for preventive therapy into clinical care.

## Collaborators

This work is on behalf of the Aspirin for Cancer Prevention Group (AsCaP): Senior Executive Board Prof. J Burn, Prof. A.T Chan, Prof. J Cuzick, Dr. B Nedjai, Prof. Ruth Langley.

## Funding

This work was supported by 10.13039/501100000289Cancer Research UK (AsCaP) [grant number C569/A24991]. KEL is supported by an 10.13039/501100000269Economic and Social Research Council studentship [grant number ES/P000745/1]. This report is independent research supported by the 10.13039/501100000272National Institute for Health Research NIHR Advanced Fellowship, Dr. Samuel Smith [grant number NIHR300588]. SGS also acknowledges funding support from a Yorkshire Cancer Research University Academic Fellowship. The funders had no role in study design, data collection and analysis, decision to publish, or preparation of the manuscript.

## Disclosure

RJT has received honorarium from Novartis. All remaining authors declare no conflicts of interest.

## Declaration of Competing Interest

The authors declare that they have no known competing financial interests or personal relationships that could have appeared to influence the work reported in this paper.

## Appendix A Supplementary data

Supplementary material 1 Image 1

Supplementary material 2 Image 2
