# Supplementary material for: Aspirin use for cancer prevention: A systematic review of public, patient and healthcare provider attitudes and adherence behaviours
Source: Prev Med. 2022 Jan;154:106872. doi: 10.1016/j.ypmed.2021.106872 (PMC8803547; doi:10.1016/j.ypmed.2021.106872)
Supplement: Supplementary file 1 — Supplementary material 1 [file mmc1.docx]

**Supplementary Table 1. Characteristics of articles reporting all participant uptake rates to a clinical trial involving the use of aspirin for cancer prevention (*n* = 4)**

| **Study** | **Country** | **Design and quality** | **Population** | **Dose/timing** | ***n^*^*** | **Age, years** | **All participant trial uptake^**^** |
| --- | --- | --- | --- | --- | --- | --- | --- |
| Hull et al. 2018^30^ | UK | RCT  *MMAT Score:* 4 | Higher risk patients with colorectal adenomas | 300mg/daily and/ or eicosapentaenoic acid | 709 | Mean: 65 | 18.1% |
| Jankowski et al. 2018^31^ | UK and Canada | RCT  *MMAT Score:* 2 | Patients with Barrett’s oesophagus | 300mg/daily (UK) or 325mg/daily (Canada) plus esomeprazole | 2,557 | Mean: 58-59 | 44.7% |
| Logan et al.  2008^28^ | UK | RCT  *MMAT Score:* 3 | Higher risk patients with colorectal adenomas | 300mg/daily or 300mg plus folate/daily | 939 | Mean (range): 57.8 (27.6–74.6) | 13.3% |
| Rexrode et al. 2000^29^ | US | RCT  *MMAT Score:* 1 | Women healthcare providers aged ≥45 | 100mg/alternate day plus vitamin E | 39,876 | 45-54 (60.2%); 55-64 (29.5%);  >65 (10.3%) | 3.7%*** |
|  |  |  |  |  |  |  | 14.4%**** |

Key: RCT = Randomised Control Trial; MMAT = Mixed Methods Appraisal Tool; *n^*^ =* number of participants enrolled at the beginning of the study; All participant trial uptake** = proportion of individuals who enrolled on the trial, with denominator number of people offered the trial. 3.7%*** = proportion of people who took part in the trial run-in placebo, with denominator the number of people who were sent a questionnaire informing them about the trial. 14.4%**** = proportion of people who took part in the trial run-in placebo, with denominator the number of people who returned the questionnaire.
