# Supplementary material for: Aspirin use for cancer prevention: A systematic review of public, patient and healthcare provider attitudes and adherence behaviours
Source: Prev Med. 2022 Jan;154:106872. doi: 10.1016/j.ypmed.2021.106872 (PMC8803547; doi:10.1016/j.ypmed.2021.106872)
Supplement: Supplementary file 2 — Supplementary material 2 [file mmc2.docx]

Search Strategies

In March 2018 we searched for studies examining the use of aspirin to prevent the development of cancer. We updated and re-ran the searches in February 2020. The following databases and websites were searched:

| **Database** | **Date Searched** | Searcher |
| --- | --- | --- |
| Cancer.gov | 24/2/20 | NK |
| Cancer Research UK | 24/2/20 | NK |
| CINAHL (EBSCO) 1981- present | 20/2/20 | NK |
| ClinicalTrials.gov (U.S. NIH) | 20/2/20 | NK |
| Cochrane Central Register of Controlled Trials (Wiley): Issue 2 of 12, February 2020 | 20/2/20 | NK |
| Cochrane Database of Systematic Reviews (Wiley): Issue 2 of 12, February 2020 | 20/2/20 | NK |
| Database of Abstracts of Reviews of Effect (Wiley) : Issue 2 of 4, April 2015 | 27/3/18 | RR |
| CRD HTA database only all available dates (using Pan Canadian interface) | 20/2/20 | NK |
| Dissertations & Theses A&I (Proquest) 1743 – present | 20/2/20 | NK |
| Embase Classic+Embase (Ovid) 1947 to 2020 February 19 | 20/2/20 | NK |
| Health Technology Assessment Database (Wiley): Issue 4 of 4, October 2016 | 27/3/18 | RR |
| International Clinical Trials Registry Platform (WHO) | 20/2/20 | NK |
| Ovid MEDLINE(R) and Epub Ahead of Print, In-Process & Other Non-Indexed Citations and Daily 1946 to February 19, 2020 | 20/2/20 | NK |
| NHS Economic Evaluation Database (Wiley): Issue 2 of 4, April 2015 | 27/3/18 | RR |
| PubMed (NLM) 1946 – present | 27/3/18 | RR |
| Web of science core collection:   - Science Citation Index Expanded (SCI-EXPANDED) --1900-present - Social Sciences Citation Index (SSCI) --1900-present - Arts & Humanities Citation Index (A&HCI) --1975-present - Conference Proceedings Citation Index- Science (CPCI-S) --1990-present - Conference Proceedings Citation Index- Social Science & Humanities (CPCI-SSH) --1990-present - Emerging Sources Citation Index (ESCI) --2015-present | 20/2/20 | NK |
| **Total records: 17,344**  **After duplicated records were removed by EndNote and Covidence: 11,258 (1662 unique in 2020 update)** |  |  |

## Cancer.gov

https://www.cancer.gov/about-cancer/treatment/clinical-trials/search

Date searched: 24/2/20

Aspirin selected 1/13 (2 were unique and 1 was not relevant)
Acetylsalicylic Acid 0/1 (0 unique)

## Cancer Research UK

http://www.cancerresearchuk.org/about-cancer/find-a-clinical-trial

Date searched: 24/2/20

Aspirin 0/1 unique hits
Acetylsalicylic Acid 0/0

## CINAHL (EBSCO) 1981- present

Thursday, February 20, 2020 10:48:29 AM

# Query Results

S11 S7 AND S10 945

S10 S8 OR S9 16,134

S9 TX (Aspirin* or "2-(Acetyloxy)benzoic Acid*" or "Acetylsalicylic Acid*" or Acetysal* or Acylpyrin* or Aloxiprimum* or Colfarit* or Dispril* or Easprin* or Ecotrin* or Endosprin* or Magnecyl* or Micristin* or Polopirin* or Polopiryna* or Solprin* or Solupsan* or Zorprin* or R16CO5Y76E or 50-78-2 or 200-064-1) 16,134

S8 (MH "Aspirin") 10,608

S7 S3 AND S6 82,381

S6 S4 OR S5 845,708

S5 TX (chemoprevent* or Chemoprophyl*) 5,535

S4 TX (prevent* or Prophyla*) 844,419

S3 ((TX (adenocarcinoma* or adenosarcoma* or angiosarcoma* or astrocytoma* or blastoma* or cancer* or carcino* or Cholangiocarcinoma* or Craniopharyngioma* or chondrosarcoma* or Ependymoma* or Fibrosarcoma* or Glioblastoma* or glioma* or Hemangioendothelioma* or Hepatoblastoma* or Hodgkin* or Leiomyosarcoma* or leuk#emia or Liposarcoma* or "Lynch Syndrome#" or lymphoma* or malignan* or Medulloblastoma* or melanoma* or Meningioma* or Mesenchymous* or Mesothelioma* or metast* or microcytic* or "Mycosis) AND (S1 OR S2)) AND (S1 OR S2) 555,949

S2 TX (adenocarcinoma* or adenosarcoma* or angiosarcoma* or astrocytoma* or blastoma* or cancer* or carcino* or Cholangiocarcinoma* or Craniopharyngioma* or chondrosarcoma* or Ependymoma* or Fibrosarcoma* or Glioblastoma* or glioma* or Hemangioendothelioma* or Hepatoblastoma* or Hodgkin* or Leiomyosarcoma* or leuk#emia or Liposarcoma* or "Lynch Syndrome#" or lymphoma* or malignan* or Medulloblastoma* or melanoma* or Meningioma* or Mesenchymous* or Mesothelioma* or metast* or microcytic* or "Mycosis 539,423

S1 (MH "Neoplasms+") 494,850

## ClinicalTrials.gov (U.S. NIH)

Date searched: 20/2/20

*condition or disease:* adenocarcinoma OR adenosarcoma OR angiosarcoma OR astrocytoma OR blastoma OR cancer OR carcinoma OR Cholangiocarcinoma OR Craniopharyngioma OR chondrosarcoma

AND
*other terms:* Aspirin OR benzoic Acid OR Acetylsalicylic Acid OR Acetysal OR Acylpyrin OR Aloxiprimum OR Colfarit OR Dispril OR Easprin OR Ecotrin OR Endosprin OR Magnecyl OR Micristin OR Polopirin OR Polopiryna OR Solprin **178**

---

*condition or disease:* Fibrosarcoma OR Glioblastoma OR glioma OR Hemangioendothelioma OR Hepatoblastoma OR Hodgkin OR Leiomyosarcoma OR leukemia OR leukaemia OR Liposarcoma OR Lynch Syndrome OR lymphoma

AND
*other terms* : Aspirin OR benzoic Acid OR Acetylsalicylic Acid OR Acetysal OR Acylpyrin OR Aloxiprimum OR Colfarit OR Dispril OR Easprin OR Ecotrin OR Endosprin OR Magnecyl OR Micristin OR Polopirin OR Polopiryna OR Solprin **10**

----

*condition or disease:* malignancy OR Malignancies OR Medulloblastoma OR melanoma OR Meningioma OR Mesenchymous OR Mesothelioma OR metastasis OR metaplasia OR Metaneoplasia OR microcytic* OR Mycosis Fungoides

AND
*other terms* : Aspirin OR benzoic Acid OR Acetylsalicylic Acid OR Acetysal OR Acylpyrin OR Aloxiprimum OR Colfarit OR Dispril OR Easprin OR Ecotrin OR Endosprin OR Magnecyl OR Micristin OR Polopirin OR Polopiryna OR Solprin **216**

*condition or disease:* Myelodysplastic OR myeloma OR neoplasia OR Neoplasm OR nephroblastoma OR Neuroblastoma OR Non-Hodgkin OR Oligodendroglioma OR oncology OR Oncogenic OR Osteosarcoma OR Pancreatoblastoma
AND
*other terms* : Aspirin OR benzoic Acid OR Acetylsalicylic Acid OR Acetysal OR Acylpyrin OR Aloxiprimum OR Colfarit OR Dispril OR Easprin OR Ecotrin OR Endosprin OR Magnecyl OR Micristin OR Polopirin OR Polopiryna OR Solprin **177**

*condition or disease:* Paget OR Pheochromocytoma OR Pineoblastoma OR retinoblastoma OR Rhabdomyosarcoma OR sarcoma OR teratoma OR tumor OR tumour OR Thymoma

AND
*other terms* : Aspirin OR benzoic Acid OR Acetylsalicylic Acid OR Acetysal OR Acylpyrin OR Aloxiprimum OR Colfarit OR Dispril OR Easprin OR Ecotrin OR Endosprin OR Magnecyl OR Micristin OR Polopirin OR Polopiryna OR Solprin **177 (same as above – double checked)**

## Cochrane Library

Date searched: 20/2/20

#1 MeSH descriptor: [Neoplasms] explode all trees and with qualifier(s): [prevention & control - PC] 4038

#2 MeSH descriptor: [Neoplasms] explode all trees 77072

#3 (adenocarcinoma* or adenosarcoma* or angiosarcoma* or astrocytoma* or blastoma* or cancer* or carcino* or Cholangiocarcinoma* or Craniopharyngioma* or chondrosarcoma* or Ependymoma* or Fibrosarcoma* or Glioblastoma* or glioma* or Hemangioendothelioma* or Hepatoblastoma* or Hodgkin* or Leiomyosarcoma* or leuk?emia or Liposarcoma* or "Lynch Syndrome?" or lymphoma* or malignan* or Medulloblastoma* or melanoma* or Meningioma* or Mesenchymous* or Mesothelioma* or metast* or microcytic* or "Mycosis Fungoides" or Myelodysplastic* or myeloma* or neoplas* or nephroblastoma* or Neuroblastoma* or Non-Hodgkin* or Oligodendroglioma* or oncolog* or Osteosarcoma* or Pancreatoblastoma* or Paget* or Pheochromocytoma* or Pineoblastoma* or retinoblastoma* or Rhabdomyosarcoma* or sarcoma* or teratoma* or tumo?r* or Thymoma*):ti,ab,kw (Word variations have been searched) 235637

#4 #2 or #3 238958

#5 MeSH descriptor: [Primary Prevention] explode all trees 3985

#6 MeSH descriptor: [Secondary Prevention] explode all trees 3069

#7 MeSH descriptor: [Chemoprevention] explode all trees 1614

#8 (prevent* or Prophyla*):ti,ab,kw (Word variations have been searched) 235695

#9 (chemoprevent* or Chemoprophyl*):ti,ab,kw 3026

#10 #5 or #6 or #7 or #8 or #9 237553

#11 (#4 and #10) 34282

#12 #11 or #1 34282

#13 MeSH descriptor: [Aspirin] explode all trees 5764

#14 (Aspirin* or "2-(Acetyloxy)benzoic Acid*" or "Acetylsalicylic Acid*" or Acetysal* or Acylpyrin* or Aloxiprimum* or Colfarit* or Dispril* or Easprin* or Ecotrin* or Endosprin* or Magnecyl* or Micristin* or Polopirin* or Polopiryna* or Solprin* or Solupsan* or Zorprin* or R16CO5Y76E or "50-78-2" or "200-064-1"):ti,ab,kw 16423

#15 #13 or #14 16423

#16 #12 AND #15 679

- Cochrane Database of Systematic Reviews: Issue 2 of 12, February 2020 (6)
- Cochrane Central Register of Controlled Trials: Issue 2 of 12, February 2020 (669)
- Did not download editorial (1)

## CRD HTA database all available dates

Date searched: 20/2/20

1 MeSH DESCRIPTOR Neoplasms EXPLODE ALL TREES 11971

2 (adenocarcinoma* or adenosarcoma* or angiosarcoma* or astrocytoma* or blastoma* or cancer* or carcino* or Cholangiocarcinoma* or Craniopharyngioma* or chondrosarcoma* or Ependymoma* or Fibrosarcoma* or Glioblastoma* or glioma* or Hemangioendothelioma* or Hepatoblastoma* or Hodgkin* or Leiomyosarcoma* or leuk?emia or Liposarcoma* or "Lynch Syndrome?" or lymphoma* or malignan* or Medulloblastoma* or melanoma* or Meningioma* or Mesenchymous* or Mesothelioma* or metast* or microcytic* or "Mycosis Fungoides" or Myelodysplastic* or myeloma* or neoplas* or nephroblastoma* or Neuroblastoma* or Non-Hodgkin* or Oligodendroglioma* or oncolog* or Osteosarcoma* or Pancreatoblastoma* or Paget* or Pheochromocytoma* or Pineoblastoma* or retinoblastoma* or Rhabdomyosarcoma* or sarcoma* or teratoma* or tumo?r* or Thymoma*) IN HTA 3690

3 #1 OR #2 12884

4 MeSH DESCRIPTOR Primary Prevention EXPLODE ALL TREES 914

5 MeSH DESCRIPTOR Secondary Prevention EXPLODE ALL TREES 441

6 (prevent* or Prophyla*) IN HTA 2119

7 MeSH DESCRIPTOR Chemoprevention EXPLODE ALL TREES 382

8 (chemoprevent* or Chemoprophyl*) IN HTA 15

9 #4 OR #5 OR #6 OR #7 OR #8 3712

10 #3 AND #9 537

11 MeSH DESCRIPTOR Aspirin EXPLODE ALL TREES 387

12 (Aspirin* ) IN HTA 77

13 (Acetylsalicylic Acid* ) IN HTA 13

14 (Acetysal* or Acylpyrin* or Aloxiprimum* or Colfarit* or Dispril* or Easprin* or Ecotrin* or Endosprin* or Magnecyl* or Micristin* or Polopirin* or Polopiryna* or Solprin* or Solupsan* or Zorprin* ) IN HTA 1

15 #11 OR #12 OR #13 OR #14 440

16 #10 AND #15 17

## Database of Abstracts of Reviews of Effect (Wiley): Issue 2 of 4, April 2015

Date searched: 27/3/18

See Cochrane strategy

Number retrieved = 22

## Dissertations & Theses A&I (Proquest) 1743 – present

Date searched: 20/2/20

ti((adenocarcinoma* or adenosarcoma* or angiosarcoma* or astrocytoma* or blastoma* or cancer* or carcino* or Cholangiocarcinoma* or Craniopharyngioma* or chondrosarcoma* or Ependymoma* or Fibrosarcoma* or Glioblastoma* or glioma* or Hemangioendothelioma* or Hepatoblastoma* or Hodgkin* or Leiomyosarcoma* or leuk?emia or Liposarcoma* or "Lynch Syndrome?" or lymphoma* or malignan* or Medulloblastoma* or melanoma* or Meningioma* or Mesenchymous* or Mesothelioma* or metast* or microcytic* or "Mycosis Fungoides" or Myelodysplastic* or myeloma* or neoplas* or nephroblastoma* or Neuroblastoma* or Non-Hodgkin* or Oligodendroglioma* or oncolog* or Osteosarcoma* or Pancreatoblastoma* or Paget* or Pheochromocytoma* or Pineoblastoma* or retinoblastoma* or Rhabdomyosarcoma* or sarcoma* or teratoma* or tumo?r* or Thymoma*) AND (Aspirin* or "2-(Acetyloxy)benzoic Acid*" or "Acetylsalicylic Acid*" or Acetysal* or Acylpyrin* or Aloxiprimum* or Colfarit* or Dispril* or Easprin* or Ecotrin* or Endosprin* or Magnecyl* or Micristin* or Polopirin* or Polopiryna* or Solprin* or Solupsan* or Zorprin* or R16CO5Y76E or 50-78-2 or 200-064-1)) OR ab((adenocarcinoma* or adenosarcoma* or angiosarcoma* or astrocytoma* or blastoma* or cancer* or carcino* or Cholangiocarcinoma* or Craniopharyngioma* or chondrosarcoma* or Ependymoma* or Fibrosarcoma* or Glioblastoma* or glioma* or Hemangioendothelioma* or Hepatoblastoma* or Hodgkin* or Leiomyosarcoma* or leuk?emia or Liposarcoma* or "Lynch Syndrome?" or lymphoma* or malignan* or Medulloblastoma* or melanoma* or Meningioma* or Mesenchymous* or Mesothelioma* or metast* or microcytic* or "Mycosis Fungoides" or Myelodysplastic* or myeloma* or neoplas* or nephroblastoma* or Neuroblastoma* or Non-Hodgkin* or Oligodendroglioma* or oncolog* or Osteosarcoma* or Pancreatoblastoma* or Paget* or Pheochromocytoma* or Pineoblastoma* or retinoblastoma* or Rhabdomyosarcoma* or sarcoma* or teratoma* or tumo?r* or Thymoma*) AND (Aspirin* or "2-(Acetyloxy)benzoic Acid*" or "Acetylsalicylic Acid*" or Acetysal* or Acylpyrin* or Aloxiprimum* or Colfarit* or Dispril* or Easprin* or Ecotrin* or Endosprin* or Magnecyl* or Micristin* or Polopirin* or Polopiryna* or Solprin* or Solupsan* or Zorprin* or R16CO5Y76E or 50-78-2 or 200-064-1)) 234

## Embase Classic+Embase (Ovid) 1947 to 2020 February 19

Date searched: 20/2/20

1 cancer prevention/ (41624)

2 exp Neoplasm/pc (77020)

3 exp *Neoplasm/ (3535622)

4 (adenocarcinoma* or adenosarcoma* or angiosarcoma* or astrocytoma* or blastoma* or cancer* or carcino* or Cholangiocarcinoma* or Craniopharyngioma* or chondrosarcoma* or Ependymoma* or Fibrosarcoma* or Glioblastoma* or glioma* or Hemangioendothelioma* or Hepatoblastoma* or Hodgkin* or Leiomyosarcoma* or leuk?emia or Liposarcoma* or "Lynch Syndrome?" or lymphoma* or malignan* or Medulloblastoma* or melanoma* or Meningioma* or Mesenchymous* or Mesothelioma* or metast* or microcytic* or "Mycosis Fungoides" or Myelodysplastic* or myeloma* or neoplas* or nephroblastoma* or Neuroblastoma* or Non-Hodgkin* or Oligodendroglioma* or oncolog* or Osteosarcoma* or Pancreatoblastoma* or Paget* or Pheochromocytoma* or Pineoblastoma* or retinoblastoma* or Rhabdomyosarcoma* or sarcoma* or teratoma* or tumo?r* or Thymoma*).tw,kw. (5299129)

5 or/3-4 (5699829)

6 primary prevention/ (40026)

7 secondary prevention/ (27909)

8 prevention study/ (3816)

9 (prevent* or Prophyla*).tw,kw. (2082238)

10 chemoprophylaxis/ (25231)

11 (chemoprevent* or Chemoprophyl*).tw,kw. (35882)

12 or/6-11 (2124291)

13 and/5,12 (364501)

14 or/1-2,13 (413454)

15 *acetylsalicylic acid/ (60304)

16 (Aspirin* or "2-(Acetyloxy)benzoic Acid*" or "Acetylsalicylic Acid*" or Acetysal* or Acylpyrin* or Aloxiprimum* or Colfarit* or Dispril* or Easprin* or Ecotrin* or Endosprin* or Magnecyl* or Micristin* or Polopirin* or Polopiryna* or Solprin* or Solupsan* or Zorprin* or R16CO5Y76E or 50-78-2 or 200-064-1).tw,kw,rn. (223440)

17 or/15-16 (223490)

18 and/14,17 (7602)

## Health Technology Assessment Database (Wiley): Issue 4 of 4, October 2016

Date searched: 27/3/18

See Cochrane strategy

Number retrieved = 6

## International Clinical Trials Registry Platform (WHO)

Date searched: 20/2/20

*Condition:* adenocarcinoma OR adenosarcoma OR angiosarcoma OR astrocytoma OR blastoma OR cancer OR carcinoma OR Cholangiocarcinoma OR Craniopharyngioma OR chondrosarcoma

AND
*Intervention:* Aspirin OR benzoic Acid OR Acetylsalicylic Acid OR Acetysal OR Acylpyrin OR Aloxiprimum OR Colfarit OR Dispril OR Easprin OR Ecotrin OR Endosprin OR Magnecyl OR Micristin OR Polopirin OR Polopiryna OR Solprin **288 records (for 160 trials)**

---

*Condition:* Fibrosarcoma OR Glioblastoma OR glioma OR Hemangioendothelioma OR Hepatoblastoma OR Hodgkin OR Leiomyosarcoma OR leukemia OR leukaemia OR Liposarcoma OR Lynch Syndrome OR lymphoma

AND
*Intervention:* Aspirin OR benzoic Acid OR Acetylsalicylic Acid OR Acetysal OR Acylpyrin OR Aloxiprimum OR Colfarit OR Dispril OR Easprin OR Ecotrin OR Endosprin OR Magnecyl OR Micristin OR Polopirin OR Polopiryna OR Solprin **273 (for 127 trials)**

*Condition:* malignancy OR Malignancies OR Medulloblastoma OR melanoma OR Meningioma OR Mesenchymous OR Mesothelioma OR metastasis OR metaplasia OR Metaneoplasia OR microcytic* OR Mycosis Fungoides

AND
*Intervention:* Aspirin OR benzoic Acid OR Acetylsalicylic Acid OR Acetysal OR Acylpyrin OR Aloxiprimum OR Colfarit OR Dispril OR Easprin OR Ecotrin OR Endosprin OR Magnecyl OR Micristin OR Polopirin OR Polopiryna OR Solprin **284 (for 152 trials)**

*Condition:* Myelodysplastic OR myeloma OR neoplasia OR Neoplasm OR nephroblastoma OR Neuroblastoma OR Non-Hodgkin OR Oligodendroglioma OR oncology OR Oncogenic OR Osteosarcoma OR Pancreatoblastoma
AND
*Intervention:* Aspirin OR benzoic Acid OR Acetylsalicylic Acid OR Acetysal OR Acylpyrin OR Aloxiprimum OR Colfarit OR Dispril OR Easprin OR Ecotrin OR Endosprin OR Magnecyl OR Micristin OR Polopirin OR Polopiryna OR Solprin **140 (for 90 trials)**

*Condition:* Paget OR Pheochromocytoma OR Pineoblastoma OR retinoblastoma OR Rhabdomyosarcoma OR sarcoma OR teratoma OR tumor OR tumour OR Thymoma

AND
*Intervention:* Aspirin OR benzoic Acid OR Acetylsalicylic Acid OR Acetysal OR Acylpyrin OR Aloxiprimum OR Colfarit OR Dispril OR Easprin OR Ecotrin OR Endosprin OR Magnecyl OR Micristin OR Polopirin OR Polopiryna OR Solprin **356** **(for227 trials)**

## Ovid MEDLINE(R) and Epub Ahead of Print, In-Process & Other Non-Indexed Citations and Daily <1946 to February 19, 2020>

Date searched: 20/2/20

1 exp Neoplasm/pc (94259)

2 exp Neoplasm/ (3285261)

3 (adenocarcinoma* or adenosarcoma* or angiosarcoma* or astrocytoma* or blastoma* or cancer* or carcino* or Cholangiocarcinoma* or Craniopharyngioma* or chondrosarcoma* or Ependymoma* or Fibrosarcoma* or Glioblastoma* or glioma* or Hemangioendothelioma* or Hepatoblastoma* or Hodgkin* or Leiomyosarcoma* or leuk?emia or Liposarcoma* or "Lynch Syndrome?" or lymphoma* or malignan* or Medulloblastoma* or melanoma* or Meningioma* or Mesenchymous* or Mesothelioma* or metast* or microcytic* or "Mycosis Fungoides" or Myelodysplastic* or myeloma* or neoplas* or nephroblastoma* or Neuroblastoma* or Non-Hodgkin* or Oligodendroglioma* or oncolog* or Osteosarcoma* or Pancreatoblastoma* or Paget* or Pheochromocytoma* or Pineoblastoma* or retinoblastoma* or Rhabdomyosarcoma* or sarcoma* or teratoma* or tumo?r* or Thymoma*).tw,kw. (3775592)

4 or/2-3 (4420303)

5 Primary Prevention/ (18118)

6 Secondary Prevention/ (19882)

7 (prevent* or Prophyla*).tw,kw. (1492889)

8 Chemoprevention/ (5870)

9 (chemoprevent* or Chemoprophyl*).tw,kw. (27070)

10 or/5-9 (1525518)

11 and/4,10 (251231)

12 or/1,11 (307565)

13 exp Aspirin/ (44354)

14 (Aspirin* or "2-(Acetyloxy)benzoic Acid*" or "Acetylsalicylic Acid*" or Acetysal* or Acylpyrin* or Aloxiprimum* or Colfarit* or Dispril* or Easprin* or Ecotrin* or Endosprin* or Magnecyl* or Micristin* or Polopirin* or Polopiryna* or Solprin* or Solupsan* or Zorprin* or R16CO5Y76E or 50-78-2 or 200-064-1).tw,kw,rn. (69938)

15 or/13-14 (69938)

16 and/12,15 (2970)

## NHS Economic Evaluation Database (Wiley): Issue 2 of 4, April 2015

Date searched: 27/3/18

See Cochrane strategy

Number retrieved = 11

## PubMed (NLM) 1946 – present

Date searched: 27/3/18

Number retrieved = 1,055

Search ((("Aspirin"[Mesh]) OR (((((Aspirin*[Title/Abstract] OR "2-(Acetyloxy)benzoic Acid*"[Title/Abstract] OR "Acetylsalicylic Acid*"[Title/Abstract] OR Acetysal*[Title/Abstract] OR Acylpyrin*[Title/Abstract] OR Aloxiprimum*[Title/Abstract] OR Colfarit*[Title/Abstract] OR Dispril*[Title/Abstract] OR Easprin*[Title/Abstract] OR Ecotrin*[Title/Abstract] OR Endosprin*[Title/Abstract] OR Magnecyl*[Title/Abstract] OR Micristin*[Title/Abstract] OR Polopirin*[Title/Abstract] OR Polopiryna*[Title/Abstract] OR Solprin*[Title/Abstract] OR Solupsan*[Title/Abstract] OR Zorprin*[Title/Abstract] OR R16CO5Y76E[Title/Abstract] OR 50-78-2[Title/Abstract] OR 200-064-1)[Title/Abstract])) OR ((Aspirin*[Other Term] OR "2-(Acetyloxy)benzoic Acid*"[Other Term] OR "Acetylsalicylic Acid*"[Other Term] OR Acetysal*[Other Term] OR Acylpyrin*[Other Term] OR Aloxiprimum*[Other Term] OR Colfarit*[Other Term] OR Dispril*[Other Term] OR Easprin*[Other Term] OR Ecotrin*[Other Term] OR Endosprin*[Other Term] OR Magnecyl*[Other Term] OR Micristin*[Other Term] OR Polopirin*[Other Term] OR Polopiryna*[Other Term] OR Solprin*[Other Term] OR Solupsan*[Other Term] OR Zorprin*[Other Term] OR R16CO5Y76E[Other Term] OR 50-78-2[Other Term] OR 200-064-1)[Other Term])) OR ((Aspirin*[EC/RN Number] OR "2-(Acetyloxy)benzoic Acid*"[EC/RN Number] OR "Acetylsalicylic Acid*"[EC/RN Number] OR Acetysal*[EC/RN Number] OR Acylpyrin*[EC/RN Number] OR Aloxiprimum*[EC/RN Number] OR Colfarit*[EC/RN Number] OR Dispril*[EC/RN Number] OR Easprin*[EC/RN Number] OR Ecotrin*[EC/RN Number] OR Endosprin*[EC/RN Number] OR Magnecyl*[EC/RN Number] OR Micristin*[EC/RN Number] OR Polopirin*[EC/RN Number] OR Polopiryna*[EC/RN Number] OR Solprin*[EC/RN Number] OR Solupsan*[EC/RN Number] OR Zorprin*[EC/RN Number] OR R16CO5Y76E[EC/RN Number] OR 50-78-2[EC/RN Number] OR 200-064-1)[EC/RN Number])))) AND ((("Neoplasms/prevention and control"[Mesh])) OR (((("Neoplasms"[Mesh]) OR ((((adenocarcinoma*[Title/Abstract] OR adenosarcoma*[Title/Abstract] OR angiosarcoma*[Title/Abstract] OR astrocytoma*[Title/Abstract] OR blastoma*[Title/Abstract] OR cancer*[Title/Abstract] OR carcino*[Title/Abstract] OR Cholangiocarcinoma*[Title/Abstract] OR Craniopharyngioma*[Title/Abstract] OR chondrosarcoma*[Title/Abstract] OR Ependymoma*[Title/Abstract] OR Fibrosarcoma*[Title/Abstract] OR Glioblastoma*[Title/Abstract] OR glioma*[Title/Abstract] OR Hemangioendothelioma*[Title/Abstract] OR Hepatoblastoma*[Title/Abstract] OR Hodgkin*[Title/Abstract] OR Leiomyosarcoma*[Title/Abstract] OR leuk*emia[Title/Abstract] OR Liposarcoma*[Title/Abstract] OR "Lynch Syndrome*"[Title/Abstract] OR lymphoma*[Title/Abstract] OR malignan*[Title/Abstract] OR Medulloblastoma*[Title/Abstract] OR melanoma*[Title/Abstract] OR Meningioma*[Title/Abstract] OR Mesenchymous*[Title/Abstract] OR Mesothelioma*[Title/Abstract] OR metast*[Title/Abstract] OR microcytic*[Title/Abstract] OR "Mycosis Fungoides"[Title/Abstract] OR Myelodysplastic*[Title/Abstract] OR myeloma*[Title/Abstract] OR neoplas*[Title/Abstract] OR nephroblastoma*[Title/Abstract] OR Neuroblastoma*[Title/Abstract] OR Non-Hodgkin*[Title/Abstract] OR Oligodendroglioma*[Title/Abstract] OR oncolog*[Title/Abstract] OR Osteosarcoma*[Title/Abstract] OR Pancreatoblastoma*[Title/Abstract] OR Paget*[Title/Abstract] OR Pheochromocytoma*[Title/Abstract] OR Pineoblastoma*[Title/Abstract] OR retinoblastoma*[Title/Abstract] OR Rhabdomyosarcoma*[Title/Abstract] OR sarcoma*[Title/Abstract] OR teratoma*[Title/Abstract] OR tumo*r*[Title/Abstract] OR Thymoma*)[Title/Abstract])) OR ((adenocarcinoma*[Other Term] OR adenosarcoma*[Other Term] OR angiosarcoma*[Other Term] OR astrocytoma*[Other Term] OR blastoma*[Other Term] OR cancer*[Other Term] OR carcino*[Other Term] OR Cholangiocarcinoma*[Other Term] OR Craniopharyngioma*[Other Term] OR chondrosarcoma*[Other Term] OR Ependymoma*[Other Term] OR Fibrosarcoma*[Other Term] OR Glioblastoma*[Other Term] OR glioma*[Other Term] OR Hemangioendothelioma*[Other Term] OR Hepatoblastoma*[Other Term] OR Hodgkin*[Other Term] OR Leiomyosarcoma*[Other Term] OR leuk*emia[Other Term] OR Liposarcoma*[Other Term] OR "Lynch Syndrome*"[Other Term] OR lymphoma*[Other Term] OR malignan*[Other Term] OR Medulloblastoma*[Other Term] OR melanoma*[Other Term] OR Meningioma*[Other Term] OR Mesenchymous*[Other Term] OR Mesothelioma*[Other Term] OR metast*[Other Term] OR microcytic*[Other Term] OR "Mycosis Fungoides"[Other Term] OR Myelodysplastic*[Other Term] OR myeloma*[Other Term] OR neoplas*[Other Term] OR nephroblastoma*[Other Term] OR Neuroblastoma*[Other Term] OR Non-Hodgkin*[Other Term] OR Oligodendroglioma*[Other Term] OR oncolog*[Other Term] OR Osteosarcoma*[Other Term] OR Pancreatoblastoma*[Other Term] OR Paget*[Other Term] OR Pheochromocytoma*[Other Term] OR Pineoblastoma*[Other Term] OR retinoblastoma*[Other Term] OR Rhabdomyosarcoma*[Other Term] OR sarcoma*[Other Term] OR teratoma*[Other Term] OR tumo*r*[Other Term] OR Thymoma*)[Other Term])))) AND ((((("Primary Prevention"[Mesh]) OR "Secondary Prevention"[Mesh]) OR ((((prevent*[Title/Abstract] OR Prophyla*)[Title/Abstract])) OR ((prevent*[Other Term] OR Prophyla*)[Other Term]))) OR "Chemoprevention"[Mesh]) OR ((((chemoprevent*[Title/Abstract] OR Chemoprophyl*)[Title/Abstract])) OR ((chemoprevent*[Other Term] OR Chemoprophyl*)[Other Term])))))

## Web of Science Core Collection

Date searched: 20/2/20

# 7 3,382 #6 AND #5

# 6 70,891 TOPIC: ((Aspirin* or "2-(Acetyloxy)benzoic Acid*" or "Acetylsalicylic Acid*" or Acetysal* or Acylpyrin* or Aloxiprimum* or Colfarit* or Dispril* or Easprin* or Ecotrin* or Endosprin* or Magnecyl* or Micristin* or Polopirin* or Polopiryna* or Solprin* or Solupsan* or Zorprin* or R16CO5Y76E or 50-78-2 or 200-064-1))

# 5 238,573 #4 AND #1

# 4 1,785,437 #3 OR #2

# 3 33,704 TOPIC: ((((chemoprevent* or Chemoprophyl*))))

# 2 1,765,228 TOPIC: ((((prevent* or Prophyla*))))

# 1 4,038,504 TOPIC: ((((adenocarcinoma* or adenosarcoma* or angiosarcoma* or astrocytoma* or blastoma* or cancer* or carcino* or Cholangiocarcinoma* or Craniopharyngioma* or chondrosarcoma* or Ependymoma* or Fibrosarcoma* or Glioblastoma* or glioma* or Hemangioendothelioma* or Hepatoblastoma* or Hodgkin* or Leiomyosarcoma* or leuk?emia or Liposarcoma* or "Lynch Syndrome?" or lymphoma* or malignan* or Medulloblastoma* or melanoma* or Meningioma* or Mesenchymous* or Mesothelioma* or metast* or microcytic* or "Mycosis Fungoides" or Myelodysplastic* or myeloma* or neoplas* or nephroblastoma* or Neuroblastoma* or Non-Hodgkin* or Oligodendroglioma* or oncolog* or Osteosarcoma* or Pancreatoblastoma* or Paget* or Pheochromocytoma* or Pineoblastoma* or retinoblastoma* or Rhabdomyosarcoma* or sarcoma* or teratoma* or tumo?r* or Thymoma*))))

Web of Science Core Collection: Citation Indexes

- Science Citation Index Expanded (SCI-EXPANDED) --1900-present
- Social Sciences Citation Index (SSCI) --1900-present
- Arts & Humanities Citation Index (A&HCI) --1975-present
- Conference Proceedings Citation Index- Science (CPCI-S) --1990-present
- Conference Proceedings Citation Index- Social Science & Humanities (CPCI-SSH) --1990-present
- Emerging Sources Citation Index (ESCI) --2015-present

Data last updated: 2020-02-19
